# Inhalational Agent Dosing Behaviors of Anesthesia Practitioners Cause Variability in End-Tidal Concentrations at the End of Surgery and Prolonged Times to Tracheal Extubation

**DOI:** 10.7759/cureus.65527

**Published:** 2024-07-27

**Authors:** Franklin Dexter, Richard H Epstein, Vivian Ip, Anil A Marian

**Affiliations:** 1 Anesthesia, University of Iowa, Iowa City, USA; 2 Anesthesiology, Perioperative Medicine, and Pain Management, University of Miami Miller School of Medicine, Miami, USA; 3 Anesthesiology, University of Calgary, Calgary, CAN

**Keywords:** minimum alveolar concentration (mac), engineering in healthcare, behavioral operations, prolonged tracheal extubation, volatile anesthesia, end-tidal inhalational agent monitor, retrospective cohort study

## Abstract

Introduction: Prolonged times to tracheal extubation are intervals from the end of surgery to extubation ≥15 minutes. We examined why there are associations with the end-tidal inhalational agent concentration as a proportion of the age‑adjusted minimum alveolar concentration (MAC fraction) at the end of surgery.

Methods: The retrospective cohort study used 11.7 years of data from one hospital. All p‑values were adjusted for multiple comparisons.

Results: There was a greater odds of prolonged time to extubation if the anesthesia practitioner was a trainee (odds ratio 1.68) or had finished fewer than five cases with the surgeon during the preceding three years (odds ratio 1.12) (both P<0.0001). There was a greater risk of prolonged time to extubation if the MAC fraction was >0.4 at the end of surgery (odds ratio 2.66, P<0.0001). Anesthesia practitioners who were trainees and all practitioners who had finished fewer than five cases with the surgeon had greater mean MAC fractions at the end of surgery and had greater relative risks of the MAC fraction >0.4 at the end of surgery (all P<0.0001). The source for greater MAC fractions at the end of surgery was not greater MAC fractions throughout the anesthetic because the means during the case did not differ among groups. Rather, there was substantial variability of MAC fractions at the end of surgery among cases of the same anesthesia practitioner, with the mean (standard deviation) among practitioners of each practitioner's standard deviation being 0.35 (0.05) and the coefficient of variation being 71% (13%).

Conclusion: More prolonged extubations were associated with greater MAC fractions at the end of surgery. The cause of the large MAC fractions was the substantial variability of MAC fractions among cases of each practitioner at the end of surgery. That variability matches what was expected from earlier studies, both from variability among practitioners in their goals for the MAC fraction given at the start of surgical closure and from inadequate dynamic forecasting of the timing of when surgery would end. Future studies should examine how best to reduce prolonged extubations by using anesthesia machines' display of MAC fraction and feedback control of end-tidal agent concentration.

## Introduction

A prolonged time to tracheal extubation is defined as an interval from the end of surgery to extubation of 15 minutes or longer [1]. Prolonged extubations are consequential [2], based on their univariate association with immediate reintubation, respiratory treatments in the post-anesthesia care unit, and the administration of flumazenil and naloxone [3]. Anesthesiologists rate extubation times longer than 15 minutes as being poor recovery from anesthesia [4]. Prolonged extubations are associated with an average of 12.4 minutes longer from the end of surgery to the patient's operating room exit than when extubation is not prolonged (P<0.0001) [5]. Such occurrences are associated with long (greater than eight-hour) workdays (P<0.0001) [6] and with a greater probability of at least one non-anesthesia practitioner idle in the operating room waiting for extubation (P<0.0001) [7].

One purpose of the current study was to examine further the univariate and adjusted relationships between the risk of prolonged extubation and the end-tidal inhalational agent concentration as a proportion of the age-adjusted minimum alveolar concentration (i.e., MAC fraction) at the end of surgery [8,9]. Generally, the MAC fraction needed for surgical closure is considerably less than that needed to block responsiveness during the intensely painful stimulation of a surgical procedure. If the MAC fraction was comparable at the end of surgery to that throughout the case, prolonged extubation would be expected because considerable time is needed for the agent to be exhaled and the MAC fraction to decrease once the vaporizer is turned off. Recently, in a prospective observational study of one case per anesthesia practitioner, "among the N=12 cases with prolonged extubation, the mean MAC fraction achieved was 0.60 (standard error 0.10) when the surgical drapes went down, significantly larger than among the N=44 cases without prolonged extubation, 0.48 (0.21), P=0.0070" [10]. We aimed as our preliminary objective to examine the generalizability of this finding among thousands of cases.

Although the precise dose-response relationship between the MAC fraction at the end of surgery and the incidence of prolonged extubation has not been quantified, our *primary goal* was to investigate the behavioral mechanism for the large variability in the independent variable, the inhalational agent concentration at the end of surgery. In other words, although we will show the association between the undesired event and the MAC fraction at the end of surgery, our primary question was how inhalational agent concentration monitors were being used by anesthesia practitioners. High concentrations at the end of surgery are likely unnecessary, causing many prolonged extubations, based on the small observational study [10]. There is little to no feedback on the proper dose for the individual patient during general anesthesia. Rather, the appropriate MAC fraction of an inhalational anesthetic comes from scientific studies of populations of patients, mostly performed during the regulatory approval process.

Modeling studies have shown very small differences in the performance of individual anesthesiologists, nurse anesthetists, and resident physicians at preventing prolonged extubations [11]. However, prolonged extubations occur more often when the anesthesia practitioner caring for the patient at the end of surgery has not previously finished at least five cases with the surgeon within the past three years [12-14]. (This is of particular concern for neurosurgery cases [13,14].) The mechanism behind the salutary effect of experience with the surgeon is unknown but may be related to improved anticipation of the end of surgery. We hypothesized that the benefit has to do with the MAC fraction near the end of surgery achieved by the anesthesia practitioners. Prolonged extubations also occur more often when the case is finished by a trainee (e.g., anesthesiology resident) [12,15]. Why that would be so with quality supervision by anesthesiologists providing instruction is also unknown [16-18]. We hypothesized that this also relates to the end-tidal agent concentration (i.e., MAC fraction) targeted at the end of surgery.

In the current study, we test the hypotheses that when cases are finished by either trainees or anesthesia practitioners who have completed few (less than five) recent (within three years) cases with the surgeon [12], the average MAC fraction is greater at the end of surgery. If true, one potential mechanism would be using higher MAC fractions throughout the case (e.g., based on the absence of age adjustment [9] when the practitioner chose the end-tidal inhalational agent concentration). An alternative mechanism would be substantial variability among cases of the same anesthesia practitioner in the MAC fraction at the end of surgery (e.g., due to imprecise anticipation that surgery is about to end). Large variability was seen in the prospective observational study, with the standard deviation of the MAC fraction equal to 0.253 [10]. Several recent operating room management studies' results predict that, without informatics support for real-time updated case duration prediction, lack of experience with individual surgeons' workflows (e.g., from being a trainee or experienced practitioner who had not recently worked with the surgeon) would result in greater MAC fraction at the end of surgery because the anesthesia practitioner waited too long to decrease the vaporizer [19-21].

## Materials and methods

Data studied

The characteristics of the study population were deliberately kept the same as for the earlier study of trainees and anesthesia practitioners, with fewer than five cases finished with the surgeon over the previous three years [12]. However, the total study period was extended by 24 weeks (Table 1), increasing the sample size. Patients having surgery in the prone position were excluded because the three earlier studies of five or fewer cases finished with the surgeon were for patients without prone positioning [12-14]. (Inclusion of prone in the current study would also have added an extra category requiring analysis of more interactions [12].)

**Table 1:** Summary of the data, listed in the sequence used in later tables "SD" represents the sample standard deviation. "MAC" refers to altitude- and age-adjusted minimum alveolar concentration. ^a^There were 481 distinct surgeons, 218, 481, 449, and 448 among the four columns. ^b^There were 568 distinct anesthesia practitioners at the end of the cases, 380, 568, 236, and 332 among the four columns. The 236+332=568 because any trainee who graduated over the study period (e.g., student registered nurse anesthetist who stayed at the hospital as a certified registered nurse anesthetist) was assigned a new blinded identifier. ^c^The sum of the counts in the first three rows exceeds the total for all agents because a few patients received one agent for part of the case and another for the remainder. The sevoflurane rows' sample sizes are smaller because it is limited to one inhalational agent. ^d^The new data versus the original study that measured the incidence of prolonged extubations was three extra eight-week periods [12]. During those last three periods, the hospital achieved uniform use of Dräger Perseus anesthesia machines (Dräger Medical, Telford, Pennsylvania, United States). The corresponding percentages excluding the last period were, among columns, for sevoflurane, 34%, 40%, 36%, and 42%, respectively, all differences <0.9% versus using all data. For any agent, corresponding percentages were 35%, 40%, 37%, and 42%, respectively, all differences <0.9%. ^e^Another type of reversal is that of opioids, using naloxone. Among all 507,859 anesthetics at the hospital between January 2012 and January 2024, using any anesthetic including monitored anesthesia care, there were just 34 cases with the use of naloxone.

Variable	Anesthesia practitioner finished five or more cases with the surgeon^a^ in the past three years^b^ (N=23,252)	Anesthesia practitioner finished fewer than five cases with the surgeon^a^ in the past three years^b^ (N=80,421)	Nurse anesthetist or anesthesiologist (N=56,077)	Trainee, principally anesthesia resident or student registered nurse anesthetist^b^ (N=47,596)
Sevoflurane mean MAC fraction throughout case >0.6^c^	89% (20,650)	89% (71,856)	90% (50,336)	89% (42,170)
Desflurane mean MAC fraction throughout case >0.6^c^	5% (1,191)	3% (2,521)	5% (2,651)	2% (1,061)
Isoflurane mean MAC fraction throughout case >0.6^c^	10% (2,283)	11% (8,949)	9% (4,992)	13% (6,240)
Prolonged extubation, ≥15 minutes from end of surgery	21% (4,881)	24% (19,742)	19% (10,619)	29% (14,004)
American Society of Anesthesiologists base units ≥11	12% (2,849)	10% (7,842)	7% (4,050)	14% (6,641)
Time from operating room entrance to the end of surgery was at least four hours	20% (4,624)	24% (19,088)	20% (11,252)	26% (12,460)
BIS monitor used	4% (898)	3% (2,011)	3% (1,775)	2% (1,134)
Age <40 years, the age basis for adjustment of MAC fraction	44% (10,218)	35% (28,421)	40% (22,501)	34% (16,138)
Age <18 years	24% (5,535)	14% (11,213)	17% (9,791)	15% (6,957)
Age in years, mean±SD	41.4±24.7	46.9±22.9	44.2±23.3	47.4±23.3
Sevoflurane MAC fraction at the end of surgery >0.4, if sevoflurane mean MAC fraction throughout case >0.6	51% (10,570)	57% (40,872)	53% (26,816)	58% (24,626)
Sevoflurane MAC fraction at the end of surgery, if sevoflurane mean MAC fraction throughout case >0.6, mean±SD	0.494±0.395	0.525±0.377	0.506±0.387	0.533±0.374
Any agent MAC fraction at the end of surgery >0.4, if the MAC fraction throughout the case >0.6	54% (12,572)	59% (47,398)	56% (31,187)	60% (28,783)
Any agent MAC fraction at the end of surgery, if the mean MAC fraction throughout the case >0.6, mean±SD	0.511±0.377	0.536±0.363	0.520±0.373	0.543±0.359
Mean sevoflurane MAC fraction from the start through the end of surgery, among cases with sevoflurane MAC fraction throughout the case >0.6, mean±SD​	1.006±0.267	0.994±0.211	1.001±0.226	0.992±0.223
Mean agent MAC fraction from the start through the end of surgery, among cases with MAC fraction throughout the case >0.6, mean±SD	1.046±0.313	1.032±0.264	1.036±0.274	1.034±0.278
Sevoflurane MAC fraction at the end of surgery >0.6, if sevoflurane mean MAC fraction throughout case >0.6^d^	35% (7,194)	40% (28,978)	37% (18,519)	42% (17,653)
Any agent MAC fraction at the end of surgery >0.6 if the mean MAC fraction throughout the case >0.6^d^	36% (8,341)	41% (32,879)	38% (21,050)	42% (20,170)
Neostigmine used	30% (6,874)	30% (24,389)	29% (16,441)	31% (14,822)
Sugammadex used	20% (4,751)	26% (20,813)	22% (12,156)	28% (13,408)
Succinylcholine or cis-atracurium with spontaneous recovery^e^	33% (7,577)	28% (22,208)	32% (18,064)	25% (11,721)
Adult general surgery	15% (3,514)	22% (18,038)	20% (11,325)	21% (10,227)
Orthopedics	29% (6,714)	16% (12,705)	21% (12,032)	16% (7,387)
Otolaryngology	12% (2,829)	10% (7,912)	11% (5,948)	10% (4,793)
Gynecology	7% (1,565)	11% (8,871)	9% (5,272)	11% (5,164)
Neurosurgery	9% (2,170)	10% (7,653)	9% (4,806)	11% (5,017)
Urology	6% (1,296)	8% (6,285)	6% (3,645)	8% (3,936)
Ophthalmology	7% (1,627)	7% (5,943)	10% (5,592)	4% (1,978)
Dentistry	4% (832)	5% (4,350)	6% (3,248)	4% (1,934)
Pediatric surgery	7% (1,565)	3% (2,110)	4% (2,141)	3% (1,534)
Cardiothoracic surgery	4% (990)	3% (2,170)	0.2% (123)	6% (3,037)

**Figure 1:**
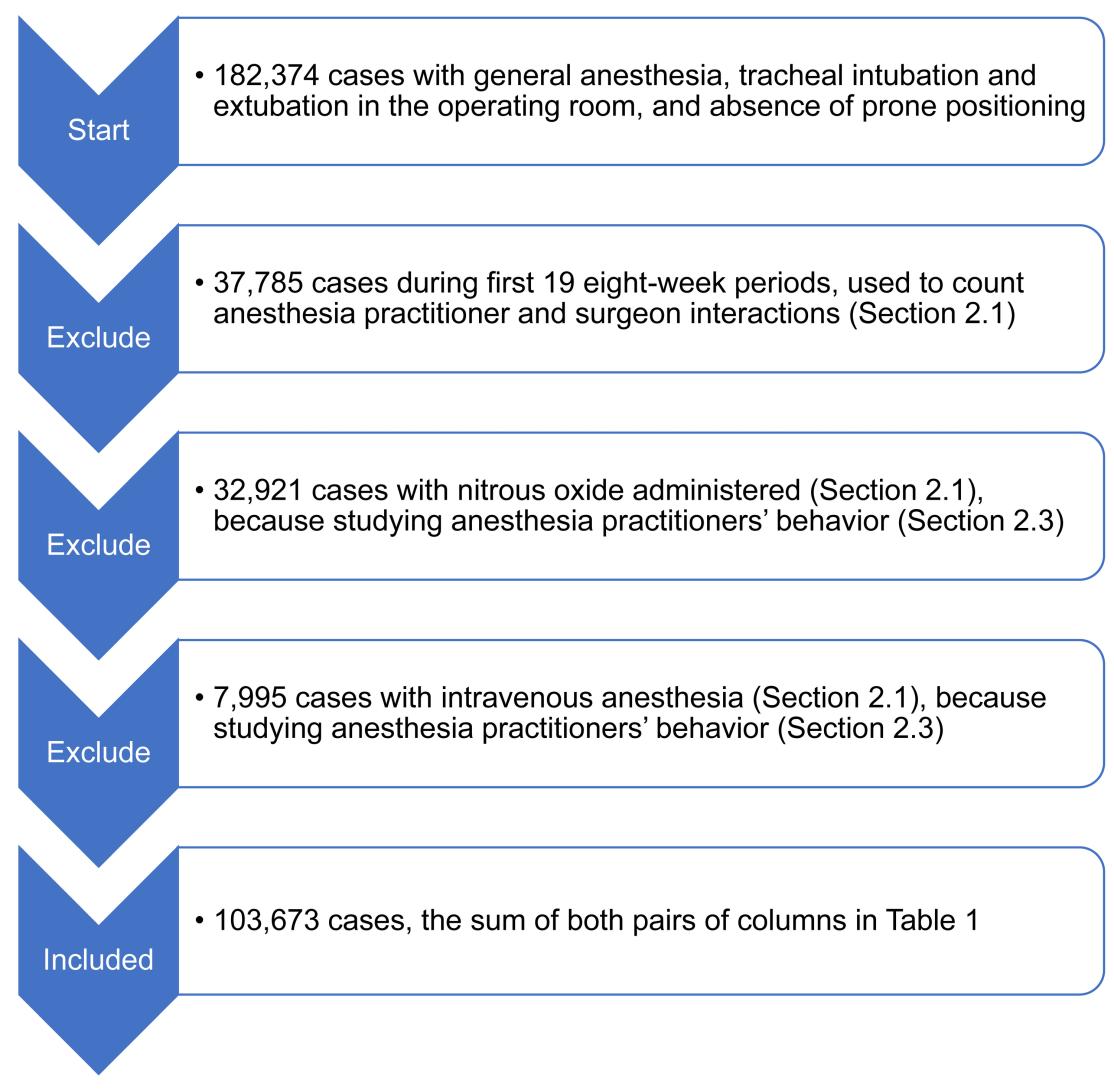
Flowchart of cases included in the study

Times of extubation were recorded using the extubation event button in the hospital's Epic electronic health record (Epic Systems, Verona, Wisconsin, United States). There were 76 whole eight-week periods from the date of implementation through Saturday 3 June 2023. That was the last period when the analyses were started. There were N=182,374 cases with general anesthesia, tracheal intubation and extubation in the operating room where the anesthetic was performed, and absence of prone positioning (Figure 1). The first 19 eight-week periods were used solely to count prior interactions between the surgeons and the anesthesia practitioners at the end of surgery (N=37,785 cases). N=32,921 more cases were excluded because nitrous oxide was administered for at least part of the case (see the third section of the Methods, "Analysis," below). Then, N=7,995 more cases were excluded for the mean MAC fraction from start through end of surgery not exceeding 0.6 because those patients received either total intravenous anesthesia or intravenous anesthesia supplemented with a low dose of an inhalational agent (e.g., MAC fraction equaled 0.4). We selected 0.6 as the threshold because it was slightly but significantly less than the MAC fraction of 0.7 needed for the reliable prevention of anesthesia awareness in the studied population lacking the use of the BIS monitor (Table 1) [22-24]. The result was that both pairs of columns in Table 1 sum to 103,673 cases (Figure 1).

Throughout the paper, all use of the abbreviation "MAC fraction" refers to end-tidal inhalational agent concentration divided by the agent's altitude- and age-adjusted minimum alveolar concentration [8,9]. The altitude adjustment of MAC is necessary because although it is almost always reported as a percentage concentration of the agent, the relevant value is partial pressure. For example, because the partial pressure at 5280 feet (1 mile) is only 480 mm Hg, compared to 760 mm Hg at sea level, a 2.1% concentration of sevoflurane in a 40‑year‑old patient in Denver, Colorado, would correspond to 1.3% in Miami, Florida. The study hospital's altitude is 204 meters above sea level, resulting in an altitude adjustment of 0.977 times the measured concentration. Age has a substantial effect, greater for ages less than 40 years and less for ages greater than 40 years (Table 1) [9]. For example, for the 75-year-old patient at the hospital, the minimum overall mean end-tidal concentration of sevoflurane studied would be 0.9%. At the study hospital, all anesthesia machines displayed the end-tidal agent concentration (Table 1). The MAC fractions were displayed when age was entered manually into the anesthesia machine.

The end of surgery was documented when the dressing was applied or when the end of surgery was noted if the time of dressing placement was not recorded (e.g., rigid bronchoscopy). The anesthesia practitioner(s) who completed the case with the surgeon was obtained from the time of extubation and the timestamps for the practitioners' start and end of care in the case staffing table. When, in addition to the anesthesiologist, there was more than one anesthesia practitioner present at the time of tracheal extubation (e.g., certified registered nurse anesthetist and a student nurse anesthetist), the one person used for counting experience with the surgeon was selected in the sequence of nurse anesthetist, anesthesiology fellow, anesthesiology resident, student nurse anesthetist, and then resident physician rotating on anesthesia (e.g., oral maxillofacial surgery). The anesthesia practitioner finishing the case was categorized as a non-trainee if licensed and credentialed by the hospital to administer anesthesia. The non-trainees were occasionally anesthesiologists who personally performed the anesthetic, but most often, they were nurse anesthetists with clinical supervision by an anesthesiologist (Table 1, third column) [25-28]. Otherwise, the practitioner was categorized as a trainee (Table 1, fourth column).

Definition of prolonged time to tracheal extubation

The choice of ≥15 minutes from the end of surgery to tracheal extubation is not arbitrary as the definition of prolonged extubation [2]. First, that criterion was previously shown to be associated with multiple adverse associations, those listed in the Introduction [2-5]. Second, an alternative threshold of five minutes was previously shown to be unsuitable because only 21% (3/14) of cases had at least one person idle at the time of tracheal extubation versus 100% (9/9) of cases with the 15-minute threshold [7]. While operating room workflows reliably were slowed by times to extubation ≥15 minutes, that was not so for increases in the extubation time when briefer (e.g., from 2.5 minutes to 7.5 minutes) [7]. The paper's associated video shows that 15 minutes is very long, with an example of a surgery resident standing at the operating room table for minutes, tapping a foot, while waiting for the patient to be extubated [7]. Third, thresholds of times at least 10 minutes were impractical because 10 minutes was less than the mean time to extubation for 30% (29/98) of surgeons [5]. In contrast, the threshold of 15 minutes was sufficiently long to exceed the mean time to extubation for 100% (98/98) of the surgeons [5]. In other words, while 10 minutes was relatively long for some surgeons' cases, that was not so for all surgeons' cases, in contrast with ≥15 minutes.

Association of inhalational agent MAC fraction at the end of surgery with prolonged extubations

The first set of analyses was performed to confirm that the independent variable of the MAC fraction at the end of surgery was associated with the dependent binary variable of prolonged time to extubation. Furthermore, we excluded cases with the use of nitrous oxide for any part of the case (Figure 1), because the inclusion of cases with nitrous oxide would have needed time-varying multivariate analyses for modeling practitioners' behavior and its association with inhalational agent concentration management (see the Limitations section in the Discussion). We, therefore, performed confirmatory baseline analyses to ensure that associations of prolonged extubations with anesthesia assignments differed negligibly from those estimated previously with no restrictions on the anesthetic [12]. Reemphasizing from the Introduction, this third section of the Methods is necessary and novel [10] but preliminary to our primary focus which was anesthesia practitioners' behavior.

We used the statistical model previously developed with similar hospital data (Table 2) [11,12]. In that model, the predictive factors for prolonged extubation were (i) cases with American Society of Anesthesiologists base units 11 or larger, (ii) cases lasting at least four hours to the end of surgery, (iii) the anesthesia practitioner not having finished at least five previous cases with the surgeon over the previous 36 months, (iv) case finished by a trainee, and (v) pediatric patient (age <18 years) [12]. The first two factors were those that were predictive in earlier machine learning models by Bayman et al. with 128 hospital data variables [11]. The latter three factors were from a recent study [12]. Not having finished at least five cases with the surgeon over 36 months was significantly more strongly associated with prolonged extubations than over 24 or 12 months [12].

**Table 2:** Addition of age-adjusted minimum alveolar concentration of sevoflurane, or any inhalational agent, at the end of surgery to the logistic regression model for prolonged time to tracheal extubation For each of the three models, the first two rows are the primary variables of interest, with subsequent rows being covariates. Models A and B have 103,673 cases with sevoflurane, desflurane, or isoflurane anesthetic and no nitrous oxide, with 481 surgeons. Model A lacks the end-tidal agent concentration at the end of surgery. Model A is the baseline from the earlier study [12]. Model C has 92,506 cases with sevoflurane anesthetic and no nitrous oxide, with 480 surgeons. The adjusted p-values are reported after applying the Holm-Bonferroni adjustment for 11 comparisons.

Model	Factor	Odds ratio	P-value	P-value adjusted	99% confidence interval
A	Anesthesia practitioner has finished fewer than five cases with the surgeon in the past three years	1.12	<0.0001	<0.0001	1.04-1.20
	Trainee, principally anesthesia resident or student registered nurse anesthetist	1.68	<0.0001	<0.0001	1.60-1.78
	American Society of Anesthesiologists base units ≥11	1.50	<0.0001	<0.0001	1.27-1.78
	Time from operating room entrance to the end of surgery was at least four hours	1.41	<0.0001	<0.0001	1.30-1.53
	Patient age <18 years	2.24	<0.0001	<0.0001	1.98-2.54
B	Anesthesia practitioner has finished fewer than five cases with the surgeon in the past three years	0.94	0.30	0.89	0.81-1.09
	Trainee, principally anesthesia resident or student registered nurse anesthetist	1.35	0.0002	0.0017	1.09-1.66
	American Society of Anesthesiologists base units ≥11	1.53	<0.0001	<0.0001	1.32-1.79
	Time from operating room entrance to the end of surgery was at least four hours	1.50	<0.0001	<0.0001	1.39-1.62
	Patient age <18 years	2.17	<0.0001	<0.0001	1.90-2.47
	End-tidal agent at end of surgery MAC fraction >0.4, age-adjusted	2.66	<0.0001	<0.0001	2.29-3.10
	Interaction: End-tidal agent at end of surgery MAC fraction >0.4 *and* anesthesia practitioner has finished fewer than five cases with the surgeon in the past three years	1.15	0.026	0.15	0.98-1.36
	Interaction: End-tidal agent at end of surgery MAC fraction >0.4 *and* trainee	1.10	0.27	>0.99	0.88-1.38
	Interaction: Anesthesia practitioner has finished fewer than five cases with the surgeon in the past three years *and* trainee	1.19	0.056	0.28	0.94-1.49
	Interaction: Three-way	0.99	0.93	0.93	0.77-1.28
	BIS monitor used	0.94	0.31	0.63	0.81-1.09
C	Anesthesia practitioner has finished fewer than five cases with the surgeon in the past three years	0.94	0.29	>0.99	0.82-1.09
	Trainee, principally anesthesia resident or student registered nurse anesthetist	1.38	<0.0001	0.0002	1.13-1.68
	American Society of Anesthesiologists base units ≥11	1.59	<0.0001	<0.0001	1.35-1.86
	Time from operating room entrance to the end of surgery was at least four hours	1.51	<0.0001	<0.0001	1.39-1.64
	Patient age <18 years	2.21	<0.0001	<0.0001	1.92-2.53
	End-tidal sevoflurane at end of surgery MAC fraction >0.4, age-adjusted	2.42	<0.0001	<0.0001	2.08-2.82
	Interaction: End-tidal sevoflurane at end of surgery MAC fraction >0.4 *and* anesthesia practitioner has finished fewer than five cases with the surgeon in the past three years	1.16	0.019	0.11	0.99-1.36
	Interaction: End-tidal sevoflurane at end of surgery MAC fraction >0.4 *and* trainee	1.02	0.81	>0.99	0.82-1.28
	Interaction: Anesthesia practitioner has finished fewer than five cases with the surgeon in the past three years *and* trainee	1.19	0.047	0.24	0.95-1.49
	Interaction: Three-way	1.02	0.82	0.82	0.80-1.31
	BIS monitor used	0.94	0.30	0.89	0.80-1.10

As done previously for modeling anesthesia practitioner and surgeon interaction [12], mixed effects logistic regression was used (Stata melogit command; StataCorp, College Station, Texas, United States). Standard errors were adjusted for clustering among the 481 surgeons' procedures using a random intercept model (Table 2) [5]. Robust variance estimation was used. We report 99% confidence intervals to allow comparison with the earlier study [12]. P‑values are reported unadjusted and after the Holm-Bonferroni adjustment for 11 comparisons, the number of variables in each of the two full logistic regression models (Table 2). Adjusted P<0.05 were treated as statistically significant.

Association of trainee and practitioner-surgeon experience with MAC at the end of surgery

One possible explanation for the increased incidence of prolonged extubation is that the patients of trainees and anesthesia practitioners who finished only a few recent cases with the surgeon have a greater median MAC fraction at the end of surgery. If that were so, and if each practitioner has a small standard deviation among their cases in the MAC fractions (Table 1), then one suitable training objective would be to educate the practitioners on how anesthesia machines flow rates and the pharmacokinetics and pharmacodynamics of inhalational anesthetics are related to the timing of patient awakening and recovery (e.g., using Gas Man (Med Man Simulations, Boston, Massachusetts, United States)) [29,30]. There were two comparative analyses: (i) practitioners having finished fewer than five cases with the surgeon over the preceding three years versus practitioners who finished at least five cases with the surgeon over the preceding three years and (ii) trainees versus not trainees. These are not four categories of practitioners because the same practitioner can change their category over time (e.g., accumulate more experience with the surgeon). Reemphasizing because it is so important, Table 1 does not show four categories but rather two separate pairs because status changes over time, and for each, the status used was as of the date of surgery. The two analyses were combined with two agent groups, all inhalational agents and sevoflurane alone. For each of these four combinations, there were five statistical tests representing different behavioral models: (a) mean MAC fraction compared using Student's t‑test with unequal variances, (b) MAC fraction values compared using two-group one-way analysis of variance with robust variance estimation and random intercept by surgeon, (c) MAC fraction values compared using the same mixed effects model along with covariates of sugammadex, neostigmine, and BIS monitor (Table 1), (d) threshold of MAC fraction >0.4 or not compared using the chi-squared test, and (e) threshold of MAC fraction >0.6 or not compared using the chi-squared test. Statistical tests (a-c) have continuous dependent variables while (d-e) have binary dependent variables. We added the threshold (e) of MAC fraction >0.6 post hoc because more than half the cases had MAC fraction >0.4 (Table 1). Each p‑value was compared with 0.05 after Holm-Bonferroni adjustment for the 20 comparisons.

For these analyses (d), we used MAC fraction >0.4 at the end of surgery because the MAC fraction of 0.4 exceeds the concentration when patients awaken [31-33]. Specifically, in greater detail, 1.0 MAC prevents movement in 50% of patients without any other analgesics or sedatives [8,31]. The MAC fraction preventing half the patients from being awake is approximately 0.62 [31]. The MAC fraction that causes half the patients to have amnesia is much lower, approximately 0.2 [32]. In clinical practice, these values are overestimates of the required concentration because other drugs affecting consciousness and response to painful stimuli have been given (e.g., benzodiazepines and opioids). The threshold for intraoperative alerts in the Michigan Awareness Control Study was 0.5 MAC [33]. Anesthesia machines display MAC fractions in units of 0.1. We used the MAC fraction of 0.4 because the patients were being awakened, and 0.4 was one displayed increment (of 0.1) less than the intraoperative alert threshold. 

Association of trainee and practitioner-surgeon experience with overall case MAC fraction

A second possible explanation for prolonged extubations is that trainees and anesthesia practitioners finishing a few recent cases with the surgeon ran higher MAC fractions during the case. That would have resulted in a larger total volume of inhalational agent to be eliminated from the patients' bodies, from considerations of uptake and distribution. Given the age distribution of the patients (Table 1), this could be due, for example, to practitioners not making age adjustments of their selected end-tidal concentrations of the inhalational agents. The mean MAC fraction was calculated for the interval from the surgical incision through the end of surgery for each inhalational anesthetic and summed. (There were some cases for which the agent was switched during the case.) The same four comparisons were made for the preceding section. For each comparison, there were two statistical tests with different behavioral models: Student's t-test with unequal variances and one-way two-group analyses of variance with robust variance estimation and random intercept by surgeon. For both tests, the dependent variable was continuous. The p‑values were reported unadjusted and also reported after Holm-Bonferroni adjustment based on the eight comparisons. There also were 99% confidence intervals calculated for mean differences in MAC fraction.

## Results

Repeating earlier work to ensure the validity of using the current data set, described in the third section of the Methods

As found for the earlier retrospective cohort study [12], there were significantly greater odds of prolonged extubation if the anesthesia practitioner finishing the case was a trainee (odds ratio 1.68, adjusted P<0.0001, Table 2). The same finding applied among all practitioners if they had finished fewer than five cases with the surgeon during the preceding three years (odds ratio 1.12, adjusted P<0.0001). Although we extended the study period, excluded cases with nitrous oxide, and excluded cases with mean MAC fractions throughout the case less than 0.6, estimated odds ratios were unchanged from the earlier study [12]. (The 99% confidence intervals from Table 2 are 1.04-1.20 for practitioner-surgeon and 1.60-1.78 for trainee. In comparison, the earlier study had 1.11-1.21 and 1.64-1.76, respectively [12].) Thus, we could validly use the current data to examine the behavioral cause of the earlier observations.

As found for the earlier prospective observational study [10], using data from all practitioners, there was a greater relative risk of prolonged extubation when the MAC fractions exceeded 0.4 at the end of surgery (odds ratio 2.66, adjusted P<0.0001, Table 2). Results were comparable when sevoflurane was the sole inhalational anesthetic used during the case (Table 2). Again, this means the current data can be used validly for our behavioral study of primary interest.

**Figure 2:**
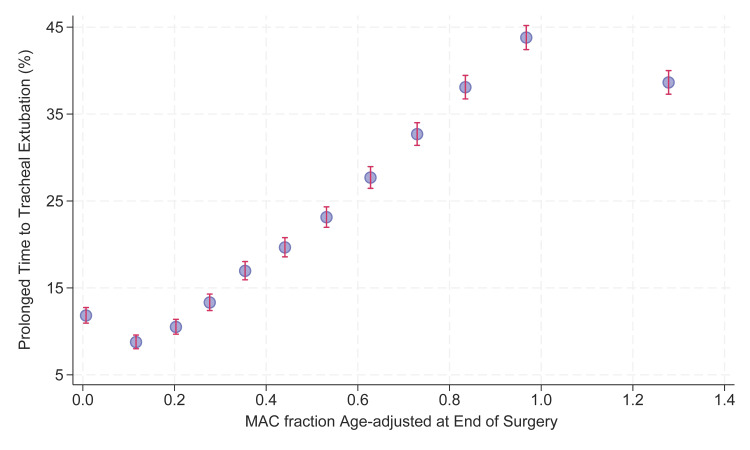
Unadjusted univariate association between the altitude- and age-adjusted minimum alveolar concentration (MAC) fraction at the end of surgery and the incidence of prolonged extubations Although the monotonic association shown was not our focus, the deviations for the two end groups have three potential explanations, likely a combination. First, the valid statistical model for the dependent variable has 10 other fixed effect parameters including the type of drug used to reverse paralysis preceding tracheal extubation, plus 481 random intercepts (Table 2). Second, toward the end of surgery, when high fresh gas flows are used, low end-tidal agent concentrations can be artifactually low. That may explain some cases in the lowest group. Third, there is a style of anesthesia referred to as deep extubation in which the agent concentration is kept at least 1.2 MAC. The patient is unconscious and breathing, but tracheal extubation is performed. For the endpoint of time from the end of surgery to tracheal extubation, that behavior would result in a small decrease in incidence, consistent with that shown in the figure in the far right-hand group.

Figure 2 shows the univariate association between the MAC fraction at the end of surgery and the incidence of prolonged extubations (i.e., 15 minutes or longer from the end of surgery to extubation). The mean MAC fractions are shown on the horizontal axis for 12 nearly equally sized groups, the size selected to have 99% Clopper-Pearson confidence intervals for the percentages approximately ±1%. (The achieved sample sizes of 8,589-8,693 cases per group are not identical because of ties.) Deviation from a monotonic association was expected given that the statistical model has 10 other fixed effect parameters and 481 random intercepts (Table 2). Nevertheless, the risk of prolonged extubation was greater for larger MAC fractions at the end of surgery, as expected (hypothesized) [2,10].

What Figure 2 shows, from a univariate perspective, is that many prolonged extubations are caused by the large MAC fractions at the end of surgery. At the studied hospital, where prolonged extubations were a sufficiently large clinical problem for the current project to be performed, once the surgery was completed, 38% (39,386/103,673) of the cases had MAC fractions greater than that needed to prevent awareness. Such a figure has not been published previously [2,10]. The comparison of MAC fraction between 12 cases with and 44 cases without prolonged extubation was recently published [10], while Figure 2 uses 103,673 cases.

The primary question in the current article, addressed below, is why there is such large variability along the horizontal axis, >250% greater than biological variability among patients [31,34]. The surgery had ended, and yet 38% (39,386/103,673) of the cases had an end-tidal concentration (horizontal axis) exceeding "MAC-awake," for which 50% of the patients would follow commands with no other drug administered [8,9,31,32]. Because the patients were anesthetized, there would be feedback to the anesthetist for the individual patient's dose for very few of the patients, such that from earlier dose-response studies [8,9,31,32], we would expect administered MAC fractions between 0.2 and 0.62.

The large standard deviations of MAC at the end of surgery were among cases of the same anesthesia practitioner, not only among anesthesia practitioners. In the recent prospective study, one observation was planned for each observed anesthesia practitioner [10]. The standard deviation among MAC fractions at the end of surgery was 0.253, N=56 [10]. Because the current study has 289 trainees and 120 non-trainees finishing at least 30 cases (Table 1), the standard deviations would be expected to be larger. All agents' end-tidal age-adjusted MAC fractions at the end of surgery had a small intraclass correlation, 0.074 (99% confidence interval 0.061-0.088). Variability could be compared between trainees and non-trainees because anesthesia practitioners did not change that status. We calculated the MAC fraction's standard deviation and quartile deviation for each practitioner at the end of surgery. The mean (SD) among practitioners of the standard deviations of MAC were 0.35 (0.04) among trainees and 0.35 (0.07) among non-trainees, larger than for the small prospective study [10], as appropriate. The mean (SD) of quartile deviations were 0.25 (0.04) among trainees and 0.24 (0.07) among non-trainees.

Results of the statistical analyses described in the fourth and fifth sections of the Methods (i.e., our primary goal)

Anesthesia practitioners who had finished fewer than five cases with the surgeon during the preceding three years, or anesthesia trainees regardless of experience with the surgeon, had greater mean MAC fractions at the end of surgery and greater relative risk of any agent MAC fraction >0.4 at the end of surgery, all adjusted P<0.0001 (Table 3). Both factors had estimated relative risk ratios of 1.09 for agent MAC fraction >0.4 at the end of surgery (Table 3). The first row of the multivariable Models A and B in Table 2 show that these increases were sufficient to account for all the associated increases in prolonged extubations from anesthesia practitioners having finished fewer than five cases within three years with the surgeon. Results were comparable for anesthetics with sevoflurane only (Table 2 and Table 3).

**Table 3:** End-tidal age-adjusted minimum alveolar concentration (MAC) fraction of sevoflurane or all inhalational anesthetics at the end of surgery CI represents confidence interval. Student's t-tests with unequal variances used Satterthwaite's degrees of freedom. The linear mixed model had standard error adjusted for clustering among 480 surgeons. The Holm-Bonferroni adjustment was based on the 25 comparisons. ^a^The large standard deviations were explained at the end of the preceding Results section. ^b^Sugammadex was associated with significantly greater MAC fraction at the end of surgery (increases 0.03, P<0.0001) and use of BIS monitor significantly lesser MAC fractions (‑0.08, P<0.0001).

Analysis	Result, all with Holm-Bonferroni adjusted P<0.0001
Association of end-tidal inhalational anesthetic at the end of surgery and anesthesia practitioner has finished *fewer* than five cases with the surgeon in the past three years versus *at least* five cases	Student's t-test with unequal variances. Mean MAC fraction (SD)^a^ increased from 0.511 (0.377) to 0.536 (0.364), the increase in mean 0.025, 99% CI 0.018-0.032
	Mixed model. Increase 0.025, 99% CI 0.012-0.037
	Mixed model including sugammadex, neostigmine, or neither administered, and BIS.^b^ Increase 0.023, 99% CI 0.011-0.034
	Chi-squared test. Risk of agent MAC fraction >0.4 increased from 54% (12572/23252) to 59% (47398/80421), risk ratio 1.09, 99% CI 1.07-1.11
	Chi-squared test. Risk of agent MAC fraction >0.6 increased from 36% (8341/23252) to 41% (32879/80421), risk ratio 1.14, 99% CI 1.11-1.17
Association of end-tidal inhalational anesthetic at end of surgery and trainee, principally anesthesia resident or student registered nurse anesthetist, versus not trainee	Student's t-test with unequal variances. Mean MAC fraction (SD)^a^ increased from 0.520 (0.373) to 0.543 (0.359), the increase in mean 0.023, 99% CI 0.017-0.029
	Mixed model. Increase 0.021, 99% CI 0.010-0.032
	Mixed model including sugammadex, neostigmine, or neither administered, and BIS.^b^ Increase 0.020, 99% CI 0.009-0.030
	Chi-squared test. Risk of agent MAC fraction >0.4 increased from 56% (31187/56077) to 60% (28783/47596), risk ratio 1.09, 99% CI 1.07-1.10
	Chi-squared test. Risk of agent MAC fraction >0.6 increased from 38% (21050/56077) to 42% (20170/47596), risk ratio 1.13, 99% CI 1.11-1.15
Association of end-tidal sevoflurane at the end of surgery and anesthesia practitioner has finished *fewer* than five cases with the surgeon in the past three years versus *at least* five cases	Student's t-test with unequal variances. Mean MAC fraction (SD)^a^ increased from 0.494 (0.395) to 0.525 (0.3477), the increase in mean 0.031, 99% CI 0.023-0.039
	Mixed model. Increase 0.027, 99% CI 0.014-0.041
	Mixed model including whether sugammadex, neostigmine, or neither administered, and BIS. Increase 0.025, 99% CI 0.012-0.038
	Chi-squared test. Risk of sevoflurane MAC fraction >0.4 increased from 51% (10570/20650) to 57% (40872/71856), risk ratio 1.11, 99% CI 1.09-1.13
	Chi-squared test. Risk of sevoflurane MAC fraction >0.6 increased from 35% (7194/20650) to 40% (28978/71856), risk ratio 1.16, 99% CI 1.13-1.19
Association of end-tidal sevoflurane at end of surgery and trainee, principally anesthesia resident or student registered nurse anesthetist, versus not trainee	Student's t-test with unequal variances. Mean MAC fraction (SD)^a^ increased from 0.456 (0.397) to 0.475 (0.399), the increase in mean 0.020, 99% CI 0.013-0.026
	Mixed model. Increase 0.030, 99% CI 0.017-0.043
	Mixed model including whether sugammadex, neostigmine, or neither administered, and BIS. Increase 0.028, 99% CI 0.015-0.040
	Chi-squared test. Risk of sevoflurane MAC fraction >0.4 increased from 53% (26816/50336) to 58% (24626/42170), risk ratio 1.10, 99% CI 1.08-1.11
	Chi-squared test. Risk of sevoflurane MAC fraction >0.6 increased from 37% (18519/50336) to 42% (17653/42170), risk ratio 1.14, 99% CI 1.11-1.16

Greater MAC fractions of the inhalational agents at the end of surgery were not a consequence of greater MAC fractions throughout the anesthetic because the means during the case were reliably no greater between groups (Table 4). The observed coefficient of variation of MAC fractions throughout the anesthetic among cases was 27%, comparable to the coefficient of variation of MAC fraction itself among patients [31,34]. In other words, dosing throughout the case was as expected. Rather, the variability in MAC fractions (horizontal axis of Figure 2) occurred while surgery was finishing.

**Table 4:** Mean end-tidal age-adjusted minimum alveolar concentration (MAC) fraction of sevoflurane or all inhalational anesthetics from the start through the end of surgery Student's t-tests with unequal variances used Satterthwaite's degrees of freedom. The mixed model with robust standard errors was adjusted for 481 clusters by the surgeons for all agents and 480 for sevoflurane. The Holm-Bonferroni adjustments were made based on the eight comparisons.

Analysis	Results
Association of end-tidal inhalational anesthetic at the end of surgery *and* anesthesia practitioner has finished *fewer* than five cases with the surgeon in the past three years versus *at least* five cases	Student's t-test with unequal variances. Mean MAC (SD) less, 1.032 (0.264, N=80,421) versus 1.046 (0.313, N=23,252). Mean decrease 0.014, adjusted P<0.0001, 99% confidence interval 0.008-0.020
	Mixed model. Mean decrease -0.001, P=0.79, adjusted P=0.79, 99% confidence interval -0.011 to 0.009
Association of mean end-tidal inhalational anesthetic *and* trainee, principally anesthesia resident or student registered nurse anesthetist	Student's t-test with unequal variances. Mean MAC (SD) less, from 1.034 (0.278, N=47,596) to 1.036 (0.274, N=56,077). Mean decrease 0.001, P=0.39, adjusted P>0.99, 99% confidence interval -0.003 to 0.006
	Mixed model. Mean decrease 0.003, P=0.41, adjusted P=0.82, 99% confidence interval -0.006 to 0.011
Association of mean end-tidal sevoflurane at the end of surgery *and* anesthesia practitioner has finished *fewer* than five cases with the surgeon in the past three years versus *at least* five cases	Student's t-test with unequal variances. Mean MAC (SD) less, 0.994 (0.211, N=71,856) versus 1.006 (0.267, N=20,650). Mean decrease 0.011, adjusted P<0.0001, 99% confidence interval 0.006-0.017
	Mixed model. Mean decrease -0.004, P=0.33, adjusted P>0.99, 99% confidence interval -0.014 to 0.006
Association of mean end-tidal sevoflurane *and* trainee, principally anesthesia resident or student registered nurse anesthetist	Student's t-test with unequal variances. Mean MAC (SD) less, 0.992 (0.223, N=42,170) versus 1.001 (0.226, N=50,336). Mean decrease 0.010, adjusted P<0.0001, 99% confidence interval 0.006-0.013
	Mixed model. Mean decrease 0.008, P=0.018, adjusted P=0.89, 99% confidence interval -0.001 to 0.016

As addressed at the end of the preceding Results section, there was substantial variability of MAC fractions at the end of surgery among cases of the same anesthesia practitioner. The mean (standard deviation) among practitioners of each practitioner's standard deviation of MAC fractions was 0.35 (0.05) (Table 1). These findings help to explain the observed behaviors shown in Table 3 and Table 4. The mean (standard deviation) among practitioners of each practitioner's coefficient of variation was 71% (13%). The observed coefficient of variation among cases was 69% (Table 1). These estimates at the end of surgery were >250% larger than biological variability in MAC fractions among patients if movement were observable [31,34].

## Discussion

Our goal was to explore end-tidal agent inhalational anesthesia monitoring by trainees, who have a substantively greater incidence of prolonged extubations, and similarly by anesthesia practitioners having finished fewer than five cases with the surgeon during the prior three years [12-14]. Our results showed that the trainees and practitioners with less prior experience with the surgeon use these clinical monitors differently. Their patients had greater MAC fractions at the end of surgery, measured multiple ways (Table 3). The magnitudes of the increases in MAC fractions were small (Table 3) but have clinical importance (Table 2). These end-of-surgery increases were not from running higher mean age-adjusted MAC fractions throughout the case (Table 4). These results were unrelated to the drugs used to reverse neuromuscular blockade after surgery (Table 3) or the use of sevoflurane (Table 1, Table 3, and Table 4), the inhalational agent most often used (Table 1). These results cannot be explained by turning the patient from a prone position, using nitrous oxide, or MAC fraction of the inhalational agent less than 0.6 throughout the case (e.g., total intravenous anesthesia), because those cases were excluded (Table 2). Given that inhalational agent concentrations are generally not titrated to just above the level where patients move (e.g., because the use of neuromuscular blockers is high and movement is too infrequent to be used for titration), variability of dose at the end of surgery >250% greater than biological variability [34,31] suggests inadequate dynamic forecasting for when the surgical drapes will be taken down. However, why would a residual association exist between being a trainee and prolonged extubation even when controlling for agent dose at the end of surgery (Table 2)? An explanation is that the trainee needs to contact the attending anesthesiologist to be present for tracheal extubation, and this allows sufficient time for the attending to reliably get there, as well as an expectation of supervision [16,17,25,27]. In contrast, at the studied hospital, the nurse anesthetists did not need to wait for the attending when they considered going ahead to be safe.

As summarized in the Introduction, prolonged extubations are associated with worse patient outcomes and greater economic costs [2]. We had hoped that examining trainees and practitioners with few cases finished with the surgeon would provide insight into how better to use end-tidal inhalation monitoring to prevent prolonged extubations when avoiding [35] drugs associated with fewer prolonged extubations, desflurane and remifentanil [1,36-38]. In other words, we cannot change the fact that there are trainees, and we cannot practically revise practitioners' assignments to cases because there is a finite set of people daily to complete the work (see the Limitations section below). We had hoped that examining the study data would, nevertheless, supply insight that applies to all practitioners. That was indeed what we learned (Figure 2). All categories of practitioners had strikingly large variability in the MAC fraction among cases at the end of surgery, including substantial frequencies of the high dose of >0.6 (Table 1 and Table 3). Variability here refers to variability among cases of the same practitioner. This observation matters behaviorally because variability among cases of each practitioner cannot be accounted for by lack of knowledge of the pharmacokinetics nor personal preferences in MAC fraction goals to prevent patient movement. (We recently studied the latter by prospective observation and asking for MAC fraction goals [10].) The variability among cases of the same practitioner being far greater than expected biologically [34,31] was what we expected from recent operating room management science (below) [19-21].

Explanation for variability in MAC fractions at the end of surgery among cases of the same practitioner

Conveniently for the quality of the current retrospective cohort study, no managerial tools [19-21] were present to assist the anesthesia practitioners in judging progress in surgical closure other than their looking at the field, relying on personal knowledge based on the category of procedure, and, potentially, for some cases, discussing progress with the surgical team.

First, the time remaining in surgical cases does not decrease like simple countdown timers [20,21,39]. For example, if the mean expected duration at the start of the case is 3.0 hours, after 2.5 hours, the mean expected time remaining is not 0.5 hours but generally approximately 1.0 hours [20,21,39]. This is a direct consequence of the probability distribution of operating room times [21,39].

Second, consider ratios of operating room time remaining in cases, specifically 0.9th and 0.5th quantiles, so that data can be pooled among many procedures [40,41]. Whereas, at the time of room entry, the median (interquartile range) of the ratio among combinations of procedure and surgeon equals 1.40 (1.31-1.54), when closing starts, the ratio is significantly greater (P<0.0001), 2.42 (2.13-2.83) [21]. Furthermore, the ratio continues to grow significantly throughout closure (P<0.0001) [21].

Third, the coefficient of variation of the interval from the start of closure to operating room exit is significantly less for the combination of procedure and surgeon than for procedure alone or for the category of procedure [20]. In other words, envision a trainee finishing a case who has previously provided anesthesia for the category of "treatment fracture … lower extremity." That is insufficient experience for the end of "open treatment of tibial shaft fracture" by a pediatric orthopedic surgeon (Table 2) [20].

Based on the current study's results, we suggest three possible interventions to mitigate the problem, but the relative values are unknown [42]. First, the anesthesia or nursing team records when closure has started [20,21]. Use the closure time mathematically to estimate the operating room time remaining [20,39], update the forecast every few minutes automatically [20,39], and incorporate extra information from the anesthesia practitioner manually based on observation and communication (e.g., waiting for the pathologist to call back with the results of the frozen section) [39,43]. Second, all available electronic data from the operating room can be used to obtain estimates of the end of surgery using machine learning methods [19]. Future study is needed because the clinical signals most closely associated with finishing sooner (e.g., the administration of sugammadex or the tracheal extubation of the patient) [19] are actions by the anesthesia practitioner and thus may not add net information for that clinician. Third, encourage deliberate conversation between the surgeon and anesthesia practitioner about when to expect dressings to be placed. While the attending surgeon may be able to articulate the precise series of milestones near the end of surgery, indicating progress toward being done, with tens of thousands of surgical procedures [19,20,44-46] and hundreds of anesthesia practitioners, there is a substantial chance of lack of recognition by the anesthesia practitioner. Among the three possible interventions, the third seems the least likely to be successful, based on the prospective observational study finding that while the MAC fraction "achieved had a moderately large correlation with the MAC goal (Pearson correlation 0.65, P<0.0001)" when closure started, "controlling for the MAC goal, there was absence of significant partial correlation of the MAC achieved at end of surgery" with frequencies of deliberate conversation (e.g., minutes remaining) [10]. Regardless, all three possible interventions are predicated on the anesthesia practitioner relying on the anesthesia machine's reported altitude- and age-adjusted MAC fraction. Practitioners may also benefit from using machines that include end-tidal feedback agent control [47].

Evaluating these potential solutions is important because strategies are needed to mitigate prolonged extubations, given that desflurane use is increasingly restricted or eliminated for its environmental impact [35]. The incidences of prolonged extubation are affected by the choice of inhalational anesthetic, approximately 184% and 352% greater with sevoflurane and isoflurane versus desflurane, respectively [1,2,36,37]. Among paired patients undergoing long-duration procedures (i.e., at least four hours), while one cohort of patients receiving neither remifentanil nor desflurane had prolonged extubations for 39% of cases, those receiving both drugs had an incidence of only 6% [38].

Limitations

We deliberately did not use statistical modeling (Table 2) to estimate the revised incidence of prolonged extubations if all trainees performed as well as non-trainees and practitioners who have recently finished a few cases with the surgeon performed as well as other practitioner-surgeon pairs. Such comparison would miss the broader point that better real-time predictions of the end of surgery may benefit all anesthesia practitioners' titration of inhalational anesthetics and neuromuscular blockers (Figure 2). When the patient of an experienced nurse anesthetist who has completed 100 cases with the surgeon over the past three years has a MAC fraction ≥0.6 at the end of surgery (Table 1 and Table 3), was that because the closure was unexpectedly quick? Alternatively, was that because the operative team was waiting for a radiologist to confirm the absence of a missing surgical needle in the field? At the study hospital, such data probably would need to be obtained by a prospective observational study randomizing the use of predictive tools for forecasting the end of surgery [19,20]. Our recent observational study suggests feasibility [10].

In an earlier study, the anesthesia practitioner having completed at least five cases with the surgeon in the past three years was a better predictor of prolonged extubations than other thresholds [12]. We are not suggesting that five cases in three years is ideal. Our primary finding from the second section of the Results was that there was a large variability of end-tidal monitored MAC fraction at the end of surgery among cases of the same anesthesia practitioner, regardless of the practitioner and surgeon's experiences together or whether the practitioner was a trainee. In other words, how familiar or not was defined did not affect our conclusions.

Although extubation time was obtained from the electronic health record, potential measurement error reliably did not affect our conclusions. First, our primary goal was to test the hypotheses that when cases were finished by either trainees or anesthesia practitioners who have completed few (less than five) recent (within three years) cases with the surgeon [12], the average MAC fractions were greater at the end of surgery. Once found to hold, we tested, as planned, whether there were higher MAC fractions throughout the case. Neither of these tests used the extubation time. Second, at the start of the results, we (quoting the section header) "repeated earlier work to ensure the validity of using the current data set." Confirmation was obtained. That earlier work did not use the time of extubation (or the start of surgical closure) obtained from the electronic health record. Third, Figure 2 shows the univariate association between the MAC fraction at the end of surgery and the incidence of prolonged extubations from the electronic health record (P<0.0001). Suppose there were relatively large measurement errors because data were from the electronic health record. That would affect the dependent variable, not the independent variable. The effect would have been no association detected, contrary to our findings.

Our results may be limited to departments like that studied (Table 1) with anesthesia trainees and anesthesia practitioners who often had little experience working with the surgeon [48,49]. Although, historically, those conditions have not applied to most hospitals nationwide [50,51], their importance may have grown with the greater use of locum practitioners who work only occasionally at a facility. Nevertheless, our goals did not include consideration of revising anesthesia practitioner and surgeon pairings (e.g., because anesthesia trainees are replaced annually by new trainees), but rather to understand the behavioral mechanism for how the inhalational monitoring is being used, and to some extent that may apply to all anesthesia clinicians. In the long term, we aim to reduce the large standard deviation in MAC fractions at the end of surgery among cases of the same practitioner (Table 1). The evaluation of potential solutions is for a future study.

Our study was performed using data from one hospital. Being in the United States, the trainees often had faculty supervising one other case simultaneously. We do not know whether results are affected by supervision quality and the faculty-to-trainee ratios [16,17,25,28]. However, we focused on understanding the sources of the greater incidence of prolonged extubations, not magnitudes. To the extent that the faculty anesthesiologist would often rely on the trainee or nurse anesthetist for notification that the surgery was about to finish, that may have inflated the incidence of prolonged extubation and thereby those effect sizes (Table 2), thus resulting in more reliable findings inferentially. We recommend relying on the associations confirmed inferentially (e.g., trainees have significantly greater mean MAC at the end of surgery, Table 3), not the specific values of the standard deviations.

At the studied hospital, anesthesiologists are sometimes present at tracheal extubation done by the experienced nurse anesthetists, and sometimes not, as permitted by state law and reflecting the hospital practice [25-28]. That accounts for the differences in incorporating MAC fraction in the logistic regression between experience with the surgeon versus trainee (Table 2). At other hospitals with all extubations potentially waiting for the anesthesiologist to arrive, if not having come to the operating room before the end of surgical closure, the impact of our results would be even *larger*.

There was little, approximately 3%, use of processed electroencephalography during the anesthetics (Table 1), logically matching that there were very few cases without moderate to high inhalational agent MAC fraction throughout the anesthetic [22-24] (Figure 1). There also was no association of BIS use with prolonged extubations (Table 2). Estimated mean differences in MAC fraction at the end of surgery between studied groups differed negligibly with or without BIS included in mixed effects models (Table 3). Our study was possible because the United States, and thus our department, lacks infusion pumps to perform closed-loop or target-control intravenous anesthesia. These unique features of our department may change Figure 1, which was not our focus. Our primary goal was to understand how the monitored end-tidal agent concentration (in MAC fraction units) was being used.

The studied hospital had 24% (24,623/103,673) prolonged extubations (Table 1), while earlier studies from two other teaching hospitals reported 15% [1,2,5,7] and a third 10% [3]. This probably reflects, in part, the low use of desflurane [1,2,35-37] (Table 1) and the exclusion of patients receiving nitrous oxide. This probably reflects also the near-zero use of opioid and sedative reversal agents (Table 1 footnote e) [52]. Specifically, the hospital with the lowest observed incidence (10%) of prolonged extubations had >114‑fold more use of naloxone than our study hospital [3]. Regardless, one important observation based on the large (24%) incidence is that prolonged extubations were not substantively caused by planned patient care during physiologically complex procedures that are a small fraction of all cases (e.g., some general thoracic surgery cases with single lung ventilation).

Finally, we examined the 82% of cases without nitrous oxide. The use of nitrous oxide is decreasing (e.g., environmental reasons) [53]. Had we included nitrous oxide in analyses, the analyses would have been converted from multivariable to time-varying multivariate because clinicians cannot be assumed to make decisions by converting from end-tidal concentrations to age-adjusted MAC.

## Conclusions

Trainees and anesthesia practitioners having finished fewer than five cases with the surgeon during the prior three years had greater MAC fractions at the end of surgery despite comparable mean MAC fractions throughout the cases. Furthermore, there was very large variability in MAC fractions at the end of surgery among cases of each anesthesia practitioner, among all practitioner groups, including many cases with MAC fractions exceeding 0.6 at the end of surgery. Earlier studies showed that these results are consistent with the behavior expected from challenges in judging how soon cases will end.
